# Preventable Pulmonary Sequelae of Measles: A Case Report of an Adult Patient With Cystic Bronchiectasis

**DOI:** 10.7759/cureus.99687

**Published:** 2025-12-20

**Authors:** Sofia Magno Pinto, Mónica Pereira, Paula G Pinto

**Affiliations:** 1 Pulmonology Department, Unidade Local de Saúde Santa Maria, Lisboa, PRT

**Keywords:** clinical case report, measles complication, non-cystic fibrosis bronchiectasis, postinfectious, vaccination coverage

## Abstract

Bronchiectasis is a chronic structural lung disease characterized by irreversible bronchial dilation and persistent productive cough. In non-cystic fibrosis cases, post-infectious etiologies must be considered. Measles, a preventable and highly contagious viral illness, can cause transient immunosuppression, predisposing individuals to secondary infections and long-term pulmonary complications, including non-cystic fibrosis bronchiectasis. Vaccination against measles significantly reduces the incidence of pulmonary complications, including pneumonia, and consequently the occurrence of bronchiectasis. Despite global vaccination efforts, with measles declared eliminated in the United States in 2000, declining vaccine coverage has led to outbreaks even in developed countries.

We present a 65-year-old non-smoking woman followed since 2009, presenting with chronic productive cough, recurrent respiratory infections, and progressive dyspnea. Initial diagnosis was bilateral cystic bronchiectasis, confirmed by high-resolution CT. The etiological workup identified bilateral cystic bronchiectasis secondary to severe measles pneumonia in childhood, confirmed serologically. The disease progressed with recurrent exacerbations, requiring multiple hospitalizations. Since 2019, she has been treated with inhaled colistimethate sodium due to chronic *Pseudomonas aeruginosa* colonization. Currently, due to disease progression with a consequent obstructive ventilatory pattern, she also requires long-term oxygen therapy and nocturnal bilevel non-invasive ventilation for chronic respiratory failure.

This case highlights the preventable long-term pulmonary consequences of measles, which, although rare, can be severe and cause significant morbidity. It reinforces the importance of maintaining sustained vaccination coverage to prevent not only acute infection but also the resurgence of post-infectious bronchiectasis. It also underscores the importance of thorough etiological investigation and highlights the increased risk of poor outcomes in older patients and those requiring oxygen therapy.

## Introduction

Bronchiectasis is a chronic structural lung disease characterized by irreversible bronchial dilation, often resulting from recurrent infection or inflammation. In non-cystic fibrosis cases, post-infectious causes are common and clinically relevant. Globally, post-infectious etiology accounts for approximately 30% of bronchiectasis cases, with measles being one of the implicated viral agents, particularly in regions with low vaccination coverage. In Europe, despite high overall vaccination rates, post-infectious causes still represent 20-30% of bronchiectasis cases, with historical measles infections remaining relevant in older adult populations who lived through the pre-vaccination era [1,2].

Measles is a highly contagious viral disease that can cause transient immunosuppression, predisposing to severe bacterial superinfections and long-term pulmonary damage. The long-term pulmonary sequelae of measles are believed to arise from the intense airway inflammation and tissue injury accompanying severe infection, which may disrupt epithelial integrity and impair mucociliary function, ultimately promoting chronic structural bronchial changes [1,3].

Registry data and cohort studies demonstrate that increasing age, frequent exacerbations, chronic *Pseudomonas aeruginosa* infection, and need for oxygen therapy are independently associated with higher mortality and morbidity. Although post-infectious bronchiectasis has no definitive cure, its management focuses on enhancing airway clearance, preventing recurrent infections, and tailoring therapeutic strategies to disease severity [3,4].

## Case presentation

We report the case of a 65-year-old non-smoking woman with no history of measles vaccination, with no other relevant medical history, followed since 2009 for bilateral suppurative bronchiectasis. According to the patient, she was diagnosed with measles-associated respiratory infection and hospitalized at approximately one year of age.

She reported recurrent respiratory infections, including episodes of pneumonia managed in an outpatient setting, without formal pulmonology follow-up until 2009, when she developed a regular productive mucopurulent cough, progressive fatigue, and dyspnea on moderate exertion.

The etiological investigation excluded immunodeficiencies, autoimmune diseases, primary ciliary dyskinesia, alpha-1 antitrypsin deficiency, and cystic fibrosis, concluding (based on a consistent clinical history and serological confirmation) positive measles IgG antibodies with a compatible clinical history of severe measles pneumonia in early childhood, confirming previous infection and supporting the diagnosis of post-measles bilateral cystic bronchiectasis.

The disease progressed with recurrent exacerbations requiring multiple hospitalizations and antibiotic courses. Since 2019, she has been treated with inhaled colistimethate sodium due to chronic *Pseudomonas aeruginosa* colonization (after unsuccessful eradication attempts with ciprofloxacin) and azithromycin since August 2023, with good tolerance. The most recent sputum culture was negative.

Her most recent hospitalization occurred in 2023 due to an exacerbation of bronchiectasis with *Pseudomonas aeruginosa *isolation and chronic global respiratory failure exacerbation. At admission, arterial blood gas (on habitual O_2_ 1 L/min) was: pH 7.368, partial pressure of carbon dioxide (pCO_2_) 53.4 mmHg, partial pressure of oxygen (pO_2_) 63.3 mmHg. Chest CT revealed multiple peripheral multilobar bronchiectases with thickened walls and fluid content, associated with several centrilobular micronodular infiltrates and scattered ground-glass opacities. She completed 14 days of piperacillin-tazobactam with progressive improvement in gas exchange, achieving at discharge: pH 7.40, pCO_2_ 44.3 mmHg, pO_2_ 70 mmHg on 1 L/min O_2_.

Currently, due to disease progression with a consequent obstructive ventilatory pattern (most recent pulmonary function test: forced vital capacity {FVC} 1.52 L/46%; forced expiratory volume in 1 second {FEV_1_} {post-BD} 0.82 L/86%; FEV_1_/FVC 62), she also requires long-term oxygen therapy and nocturnal bilevel non-invasive ventilation for chronic respiratory failure.

**Figure 1:**
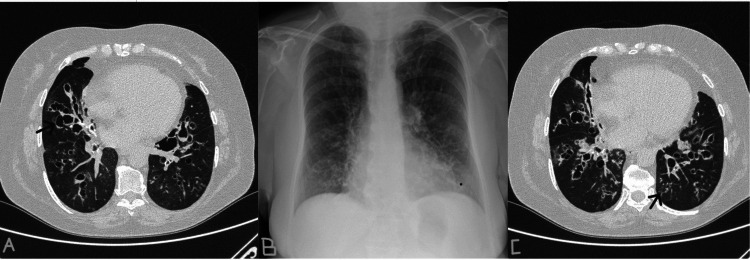
Chest CT images obtained in April 2025 (A) Axial view showing bilateral varicose-saccular bronchiectasis predominantly in the lower lobes; arrows indicate representative areas of bronchiectasis. (B) Coronal reconstruction highlighting cystic changes and bronchial wall thickening. (C) Sagittal section demonstrating associated reticulo-micronodular infiltrates suggesting chronic infectious-inflammatory changes.

Her most recent chest CT (April 2025), performed during a period of clinical stability, demonstrated bilateral varicose-saccular bronchiectasis, predominantly in the lower lobes, with associated reticulo-micronodular infiltrates consistent with chronic infectious-inflammatory changes (Figure 1). From a cardiac standpoint, no abnormalities were identified.

Written informed consent was obtained from the patient for the publication of her clinical information and imaging studies.

## Discussion

Post-measles bronchiectasis is now an uncommon but clinically significant sequela of severe childhood measles infection. Before the widespread adoption of vaccination, measles-associated pneumonia was a major cause of bronchial injury and chronic suppurative lung disease [1,2].

Post-measles bronchiectasis results from irreversible structural airway damage following severe viral infection, with destruction of the bronchial epithelium, transmural inflammation, and weakening of the airway wall leading to permanent bronchial dilation and impaired mucociliary clearance, which in turn predisposes to recurrent bacterial infections and chronic inflammation. Management follows a multifaceted approach, and in patients with three or more exacerbations per year, long-term macrolide therapy may be considered. Inhaled antibiotics may be used in selected cases, particularly in chronic *Pseudomonas aeruginosa* infection [3].

This case illustrates how incomplete vaccination coverage can allow the resurgence of preventable diseases with lasting respiratory consequences. Although the global incidence of post-measles bronchiectasis has declined, clinicians should remain aware of its possibility, particularly in older adults with a history of severe childhood pneumonia and no identifiable alternative cause [4].

Bronchiectasis can result in substantial morbidity, as observed in our patient. Older adults may have reduced mucociliary clearance, decreased immune response, and more frequent comorbidities, which can contribute to higher rates of exacerbations and mortality. It is also important to emphasize the impact of chronic *Pseudomonas aeruginosa* colonization, which is associated with a worse prognosis, increased frequency of exacerbations, and higher mortality in patients with bronchiectasis. Long-term oxygen therapy, while necessary in advanced disease, also reflects a greater degree of functional impairment and chronic respiratory failure [3,4].

## Conclusions

Measles remains a preventable cause of chronic pulmonary morbidity. This case reinforces the need for sustained global vaccination coverage to prevent both acute infection and long-term sequelae such as cystic bronchiectasis. Moreover, this report illustrates the challenges of etiological investigation in bronchiectasis, emphasizing that a careful clinical history often provides the key to uncovering the underlying cause.
